# 573. Analyzing the Effects of Pre-existing Pruritic Skin Conditions on Post-burn Outcomes

**DOI:** 10.1093/jbcr/irag033.313

**Published:** 2026-04-06

**Authors:** Mbinui Ghogomu, Yves Balikosa, Grace Obanigba, Tsola Efejuku, Ann Obi, Steven E Wolf, Michael Wilkerson, Amina El Ayadi

**Affiliations:** University of Texas Medical Branch at Galveston, Galveston, TX; University of Texas Medical Branch at Galveston, Galveston, TX; UT Houston Health Science Center, Houston, TX; University of Virginia, Charlottesville, VA; University of Texas Medical Branch at Galveston, Galveston, TX; Division of Burn and Trauma Surgery, Department of Surgery, University of Texas Medical Branch, Galveston, Galveston, TX; University of Texas Medical Branch - Department of Dermatology, Galveston, TX; Division of Burn and Trauma Surgery, Department of Surgery, University of Texas Medical Branch, Galveston, Galveston, TX

## Abstract

**Introduction:**

Burn injuries cause significant global morbidity. Although cardiovascular and renal comorbidities have been studied in relation to burns, the impact of pre-existing pruritic skin conditions is poorly understood. These conditions, including psoriasis and atopic dermatitis, are common and marked by chronic inflammation and impaired skin barriers, potentially altering post-burn outcomes. This study examined whether such disorders increase the risk of infection, scarring, irritable bowel syndrome (IBS), and depression after burn injury.

**Methods:**

Burn patients with and without pre-existing pruritic skin disease were identified in the TriNetX database. Diagnoses were based on ICD-10 codes, and cohorts were propensity-matched for age, sex, race, and ethnicity. Outcomes included infection, scarring, IBS, and depression at multiple time intervals (i.e., 2-90, 91-180, 181-365, 365+ days) following burn injury. Risk ratios with 95% confidence intervals were calculated, and significance was set at *p*<.05.

**Results:**

A total of 107 881 patients with skin disease were matched to 107 881 controls. Patients with skin disease had worse outcomes across all domains. Risk of infection was nearly doubled (RR = 1.84, *p*<.0001), scarring was modestly increased (RR = 1.31, *p*<.0001, rising to ≈2.0 after one year), and IBS risk was doubled (RR = 2.043, *p*<.0001). Depression occurred more frequently in this population, persisting across time points.

**Conclusions:**

Pre-existing pruritic skin conditions significantly worsen burn recovery, compounding risks of infection, pathological scarring, gastrointestinal dysfunction, and depression. These results suggest potential additive effects of chronic inflammatory disease and acute trauma on maladaptive healing.

**Applicability of Research to Practice:**

Identifying burn patients with pre-existing skin conditions may guide risk stratification. Enhanced infection monitoring, scar management, gastrointestinal evaluation, and psychological support could improve outcomes. Integrating dermatology, surgery, gastroenterology, and psychiatry into multidisciplinary care may reduce long-term morbidity.

**Funding for the study:**

This research was supported by an endowed fund for burn research and education, as well as by the Institute for Translational Sciences, supported in part by a Clinical and Translational Science Award from the National Center for Advancing Translational Sciences at the National Institutes of Health (NIH). The content of this study is solely the responsibility of the authors and does not necessarily represent the official views of the NIH.

